# Sulfate Activation in Mitosomes Plays an Important Role in the Proliferation of *Entamoeba histolytica*

**DOI:** 10.1371/journal.pntd.0001263

**Published:** 2011-08-02

**Authors:** Fumika Mi-ichi, Takashi Makiuchi, Atsushi Furukawa, Dan Sato, Tomoyoshi Nozaki

**Affiliations:** 1 Department of Parasitology, National Institute of Infectious Diseases, Shinjuku, Tokyo, Japan; 2 Graduate School of Medicine, Gunma University, Maebashi, Gunma, Japan; 3 Institute for Advanced Biosciences, Keio University, Tsuruoka, Yamagata, Japan; 4 Graduate School of Life and Environmental Sciences, University of Tsukuba, Tsukuba, Ibaraki, Japan; New York University School of Medicine, United States of America

## Abstract

Mitochondrion-related organelles, mitosomes and hydrogenosomes, are found in a phylogenetically broad range of organisms. Their components and functions are highly diverse. We have previously shown that mitosomes of the anaerobic/microaerophilic intestinal protozoan parasite *Entamoeba histolytica* have uniquely evolved and compartmentalized a sulfate activation pathway. Although this confined metabolic pathway is the major function in *E. histolytica* mitosomes, their physiological role remains unknown. In this study, we examined the phenotypes of the parasites in which genes involved in the mitosome functions were suppressed by gene silencing, and showed that sulfate activation in mitosomes is important for sulfolipid synthesis and cell proliferation. We also demonstrated that both Cpn60 and unusual mitochondrial ADP/ATP transporter (mitochondria carrier family, MCF) are important for the mitosome functions. Immunoelectron microscopy demonstrated that the enzymes involved in sulfate activation, Cpn60, and mitochondrial carrier family were differentially distributed within the electron dense, double membrane-bounded organelles. The importance and topology of the components in *E. histolytica* mitosomes reinforce the notion that they are not “rudimentary” or “residual” mitochondria, but represent a uniquely evolved crucial organelle in *E. histolytica.*

## Author Summary

The mitochondrion and its related organelles are ubiquitous in all extant eukaryotic cells. The mitochondria are believed to have originated from the endosymbiosis of α-proteobacteria in an ancestral eukaryote, and show diverse structures, contents, and functions. Evolution and diversification of mitochondrion-related organelles remains one of the central themes in biology. *Entamoeba histolytica*, which causes intestinal and extraintestinal amebiasis in humans, possesses a highly divergent form of mitochondrion-related organelles, named “mitosomes.” Previously, we demonstrated that sulfate activation is the major function of mitosomes in *E. histolytica*. As the sulfate activation pathway was discovered only in the cytoplasm and plastids in other eukaryotic organisms, its compartmentalization to mitosomes is unprecedented. In this study, we showed that this pathway is important for sulfolipid synthesis and cell proliferation in *E. histolytica*. Together, we infer that *E. histolytica* mitosomes are not just rudimentary or residual mitochondria, but important for proliferation of *E. histolytica*. Thus, *E. histolytica* represents a useful model to understand evolutionary constraints of mitochondrion-related organelles in eukaryotes.

## Introduction

Mitosomes and hydrogenosomes are mitochondrion-related organelles and found in a phylogenetically broad range of eukaryotes. Since organisms that possess hydrogenosomes or mitosomes do not cluster together in eukaryote phylogenies, it is suggested that secondary losses and changes in mitochondrial functions have independently occurred multiple times in eukaryote evolution [1]. This view largely agrees to the observation that the components and functions of the mitochondrion-related organelles differ between organisms [1], [2].

*Entamoeba histolytica,* a widespread intestinal protozoan parasite [3], possesses highly divergent mitosomes [4]–[6]. We have previously shown that sulfate activation is compartmentalized in *E. histolytica* mitosomes [5]. As sulfate activation generally occurs in the cytoplasm or plastids in eukaryotes [5], [7], its compartmentalization to mitosomes is unprecedented. *Mastigamoeba balamuthi*, a free-living amoeba that is distantly related to *E. histolytica,* also possesses mitochondrion-related organelle. An expressed sequence tags (EST) project showed that the organism has enzymes for sulfate activation, and one of the enzymes has the putative mitochondrial targeting signal at the amino terminus (Jan Tachezy, personal communication). *Trichomonas vaginalis, Giardia intestinalis, and Cryptosporidium parvum,* which also possess mitochondrion-related organelles, i.e., hydrogenosome and mitosome, apparently lack genes encoding these enzymes. Phylogenetic analyses further revealed that *E. histolytica* appears to have acquired enzymes involved in sulfate activation from distinct prokaryotic and eukaryotic lineages by lateral gene transfer [5]. Therefore, sulfate activation is not a common function of the mitochondrion-related organelles, but may be a unique feature of a lineage *E. histolytica* and *M. balamuthi* belong to. Although iron sulfur cluster biosynthesis is shared by aerobic eukaryotes and highly divergent *G. intestinalis* mitosomes and *T. vaginalis* hydrogenosomes [8]–[11], it still remains to be unequivocally determined whether iron sulfur cluster biosynthesis is exclusively compartmentalized to the mitosomes in *E. histolytica* and *M. balamuthi* [12].

Sulfate is generally activated in two steps. Inorganic sulfate is converted to adenosine-5′-phosphosulfate (APS), in a reaction catalyzed by ATP sulfurylase (AS), and further converted to 3′-phosphoadenosine-5′-phosphosulfate (PAPS) in a reaction catalyzed by APS kinase (APSK). Pyrophosphate concomitantly produced in the first reaction needs to be decomposed to phosphates by inorganic pyrophosphatase (IPP). PAPS acts as a sulfuryl donor to transfer the sulfuryl moiety to various acceptors by sulfotransferases, resulting into the formation of sulfurylated macromolecules such as mucopolysaccharides, sulfolipids, and sulfoproteins [7], [13], [14]. Alternatively, activated sulfate (APS and PAPS) is reduced and assimilated into cysteine [13]. In addition, activated sulfate is reduced to sulfide and used as a terminal electron acceptor in the anaerobic respiration in sulfate-reducing bacteria [15]. The *E. histolytica* genome contains 10 potential genes encoding for sulfotransferases, but lacks the enzymes for sulfate reduction [5]. Consistent with this, activated sulfate is predominantly incorporated in sulfolipids in *E. histolytica* [5]. Sulfolipids are a class of lipids containing sulfur. Among them, sulfoquinovosyldiacylglycerol and sulfolipid-I were well characterized in plastids and *Mycobacterium tuberculoris,* respectively [16], [17]. Sulfoquinovosyldiacylglycerol was shown to be involved in photosynthesis. Sulfolipid-I was identified as a virulence factor in *M. tuberculoris*. However, the structure and function of sulfolipids in *E. histolytica* remains largely unknown.

While the sulfate activation has been demonstrated as the major metabolic pathway in *E*. *histolytica* mitosomes [5], the physiological role of this mitosome-confined pathway remains unknown. In this study, we attempted to uncover the role of mitosomes in *E. histolytica* by using the parasites in which genes for the enzymes involved in sulfate activation, MCF, and Cpn60 were knocked down by gene silencing. We showed that these mitosomal proteins are indeed important for sulfolipid production and cell growth. We also demonstrated the localizations and topologies of the enzymes involved in sulfate activation, MCF, and Cpn60 in mitosomes by immunoelectron microscopy.

## Materials and Methods

### Gene silencing

G3 strain and psAP-2 plasmid were kindly given by David Mirelman, Weisman Institute, Israel [18]. An upstream region of *ap-a* gene was amplified from psAP-2 using 5′-AGCTCTAGACCGCGGCGGCTTGCTGCACCCTTTG-3′ (forward primer; *Sac*II site is underlined) and 5′-CTCTGAGCTCGTTTAAAGGCCT<$>\raster="rg1"<$>CATGATTGTTTGTAAGATAT G-3′ (reverse primer; *Sac*I, and *Stu*I sites are double-, and broken-underlined, respectively). PCR product was digested with *Sac*I and *Sac*II, and ligated into *Sac*I- and *Sac*II-double digested psAP-2 vector to produce psAP-2-Gunma. Approximately 380-450-bp fragments corresponding to the 5′ end of the open reading frame of *Cpn60, Mcf, As, Apsk,* and *Ipp* genes, respectively, were amplified by PCR using the following primers sets (*Sac*I and *Stu*I sites are single- and double-underlined, respectively):

5′-AAAGGCCTATGCTTTCATCTTCAAGTCATT-3′ (forward) and 5′-GGGGAGCTCTTTGTAATTTTCTTTAATAC-3′ (reverse) for *Cpn60*; 5′-CATCAGGCCTATGATACAAGGTATGACTTATAAACG-3′ (forward) and 5′-ACGCGAGCTCCTAGCAGTACCAAAGAATGTATC-3′ (reverse) for *Mcf*; 5′-CATCAGGCCTATGAGCATTCAAGAAAACTTAAACAAC-3′ (forward) and 5′-ACTTGAGCTCGGTCAATTTCAATAGTTCCTGAG-3′ (reverse) for *As*; 5′-GATCAGGCCTATGGCTACTGCTAAGATTGCTG-3′ (forward) and 5′-GACTGAGCTCGAGGTGGTGGTTCAACAAATTC-3′ (reverse) for *Apsk*; 5′- CATCAGGCCTATGTCAATTACTTCTATTGTCCCC-3′ (forward) and 5′-CACCGAGCTCATCAATTGGATCATTATCTCCAGG-3′ (reverse) for *Ipp*. The PCR fragments were digested with *Stu*I and *Sac*I, and ligated into the *Stu*I- and *Sac*I-double digested psAP-2-Gunma to produce the plasmids used for gene silencing. Lipofection of trophozoites and selection of transformants were performed as previously described [5].

### Production of anti-EhCpn60, EhAPSK, and EhIPP antisera

The open reading frame of *Cpn60, Apsk*, and *Ipp* was PCR-amplified with primers containing a *Bam*HI restriction site, digested with *Bam*HI, and ligated into *Bam*HI-digested pCOLD1 to yield pCOLD1-Cpn60, pCOLD1-APSK, and pCOLD1-IPP, respectively. These constructs were introduced into *E. coli* BL21 (DE3) cells. Expression and purification of the recombinant proteins were performed as previously described [19]. Briefly, *E. coli* pellet was suspended in 20 ml of lysis buffer (50 mM Tris–HCl, pH 8.0, 300 mM NaCl, and 20 mM imidazole) containing 1% Triton X-100 (v/v), 100 µg/ml lysozyme, and 25 U/ml benzonase. After 15-min incubation at 4°C, the cells were sonicated on ice and centrifuged at 12,000×*g* for 20 min at 4°C. The supernatant was applied on 50% Ni^2+^-NTA His-bind slurry (Qiagen, Tokyo, Japan). The recombinant protein-bound resin was washed three times with buffer A (50 mM Tris-HCl, pH 8.0, 300 mM NaCl) containing 20–50 mM of imidazole. The bound proteins were then eluted with buffer A containing 100 mM imidazole. Rabbit anti-Cpn60, APSK, and IPP antisera were custom made by Operon Biotechnologies (Tokyo, Japan).

### Immunoblot analysis

Whole cell lysates of each gene-silenced strain were analyzed by SDS-polyacrylamide electrophoresis (PAGE) and immunoblot analysis as previously described [5]. The dilution of the primary antibodies was 1∶1,000 for anti-Cpn60, anti-APSK, and anti-IPP antiserum, and 1∶100 for anti-CP5 antiserum [5].

### Quantitative real-time PCR (qRT-PCR)

The Fast SYBR® Green Master Mix (AB Applied Biosystems, Foster City, CA, USA) was used for qRT-PCR. RNA polymerase II gene (*Rnapol*) was used as a house-keeping reference gene. Total RNA was extracted using TRIzol® reagent (Invitrogen, Carlsbad, CA, USA). The synthesis of cDNA was performed using the SuperScript III First-Strand Synthesis System (Invitrogen). qRT-PCR was performed using the following primers: 5′-CCTATGAAAATCGATTGGACATTCTATTGCC-3′ (forward) and 5′-GCATCACCAGTAGCAAACTTTGTAACTTG-3′ (reverse) for *As*; 5′-GCCCCAATTGCACCATATCGTGAAATTAG-3′ (forward) and 5′-GCACATTGATCAACAGACTTACCAGCAG-3′ (reverse) for *Apsk*; 5′-GATCCTCTTGCTCAAAACCATTACATCTG-3′ (forward) and 5′-GTCTAACGCCAATTTTGATAACTTCTTTTGAG-3′ (reverse) for *Ipp*; 5′-GCATGTTTTGATTTTGTTGCTCCATTAGTTCC-3′ (forward) and 5′-CACTGACTAATGGAACAACTTTGACAAATCC-3′ (reverse) for *Mcf*; 5′-GATCCAACATATCCTAAAACAACA-3′ (forward) and 5′-TCAATTATTTTCTGACCCGTCTTC-3′ (reverse) for *Rnapol*. qRT-PCR was performed using StepOne Plus Real-Time PCR System (AB Applied Biosystems) with the following cycling conditions: 95°C for 20 s, followed by 40 cycles of 95°C for 3 s, and 60°C for 30 s. All reactions were run in quadruplicate, including reverse transcriptase-minus and cDNA-minus controls. Quantification for each target gene was determined by the ΔCt method with *Rnapol* as reference gene.

### Metabolic labeling

Metabolic labeling was performed as previously described [5] with some modifications. Briefly, approximately 3×10^5^ trophozoites were labeled with [^35^S]-labeled sulfate (25 mCi/mmol) in 1 mL of the BI-S-33 medium either continuously for 2, 4, or 8 h, or labeled for 4 h and chased for 4 or 24 h after removing [^35^S]-labeled sulfate. Cells were collected and lipids were extracted with 0.5 mL of methanol and separated on a silica high-performance thin-layer chromatography plate in 35∶65∶8 (vol/vol/vol) methanol/chloroform/28% (w/w) ammonium hydroxide [20]. Thin-layer chromatography plates were dried and analyzed by autoradiography.

### Immunoelectron microscopy analysis

*E. histolytica* transformants expressing epitope-tagged mitosomal proteins were previously established [5]. Approximately 5×10^5^ trophozoites were resuspended in 2 ml BI-S-33 medium and seeded onto a molybdenum disk (Nissin EM Co., JAPAN) in a well of a 24-well plate. After 15-min incubation at 35.5°C, the molybdenum disk that amoebas adhered to was removed and immediately immersed in liquid propane at −175°C. The disk was further fixed and sectioned as previously described [21]. The disk was reacted with primary antibody diluted at 1∶2000 (anti-Cpn60 antiserum) and 1∶500 (anti-HA monoclonal antibody) in phosphate-buffered saline containing 1.5% bovine serum albumin for overnight at 4°C. The samples were then reacted with colloidal gold-conjugated anti-rabbit or anti-mouse secondary antibody (1∶20) for 1 h at room temperature. Samples were examined by electron microscopy at Tokaii Microscopy., Inc (Nagoya, JAPAN).

## Results

### Establishment of gene-silenced strains

**Figure 1:**
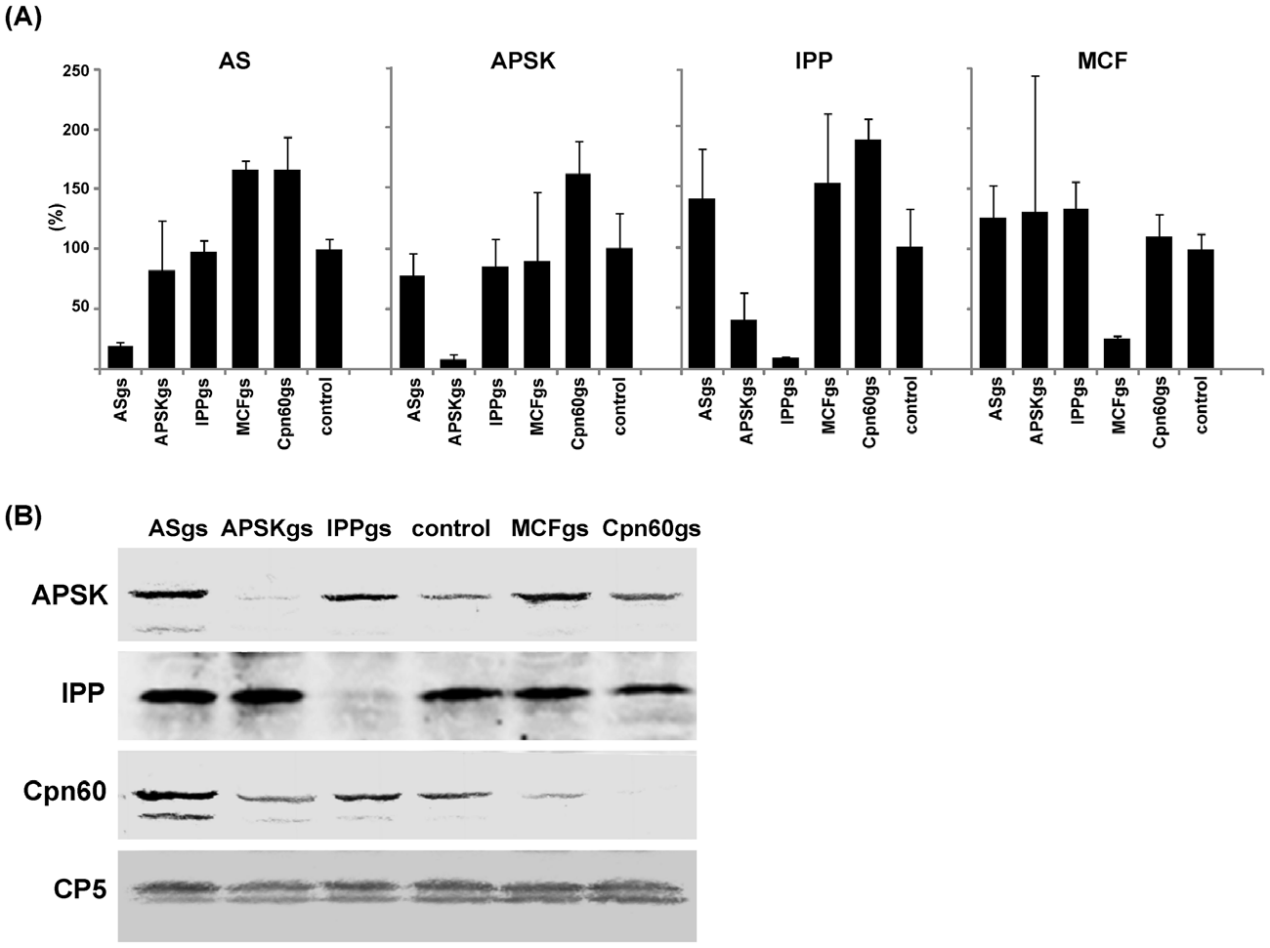
Gene silencing of AS, APSK, IPP, MCF, and Cpn60. (A) qRT-PCR analysis of AS, APSK, IPP, MCF expression in the gene-silenced strains. The relative expression level is expressed in percentage of the control strain. (B) Immunoblot analysis of the gene-silenced strains. Approximately 40 µg of lysates of the gene-silenced strains were subjected to SDS-PAGE and immunoblot analysis using anti-APSK, IPP, Cpn60, and CP5 antisera. Control, G3 strain transfected with an empty vector (psAP-2-Gunma).

To investigate the role of the mitosomes and, more specifically, mitosome-localized sulfate activation pathway, chaperones, and ADP/ATP transporter, we established the *E. histolytica* strains in which AS, APSK, IPP, MCF, and Cpn60 genes were knocked down by gene silencing [18], [22]. These gene-silenced strains were designated ASgs, APSKgs, IPPgs, MCFgs, and Cpn60gs strains, respectively. AS, APSK, and IPP are essential components of the sulfate activation pathway, while MCF transports ADP/ATP across the mitosomal membrane and Cpn60 functions as a mitosome-specific chaperone [5]. Reduction of gene expression of each target gene was verified by qRT-PCR in the gene-silenced strains. The amount of the steady-state transcript of the genes involved in sulfate reduction was reduced by 80.4–91.8% (Figure 1A). The changes of the level of the transcripts of irrelevant genes ranged 0.4–1.8 fold of the control, but mostly varied only in the range of 0.8–1.6 fold. In APSKgs, IPPgs, and Cpn60gs strains, the reduction of each target protein was confirmed by immunoblotting (Figure 1B). Although we observed slight variations in the amount of APSK and Cpn60 in the gene-silenced strains (e.g., reduction of Cpn60 protein in MCFgs strain), these variations did not correlate with the changes in the transcripts. In APSKgs strain, IPP mRNA level was also slightly decreased, while its protein level remained unchanged.

### Effects of repression of sulfate activation on sulfolipid synthesis

**Figure 2:**
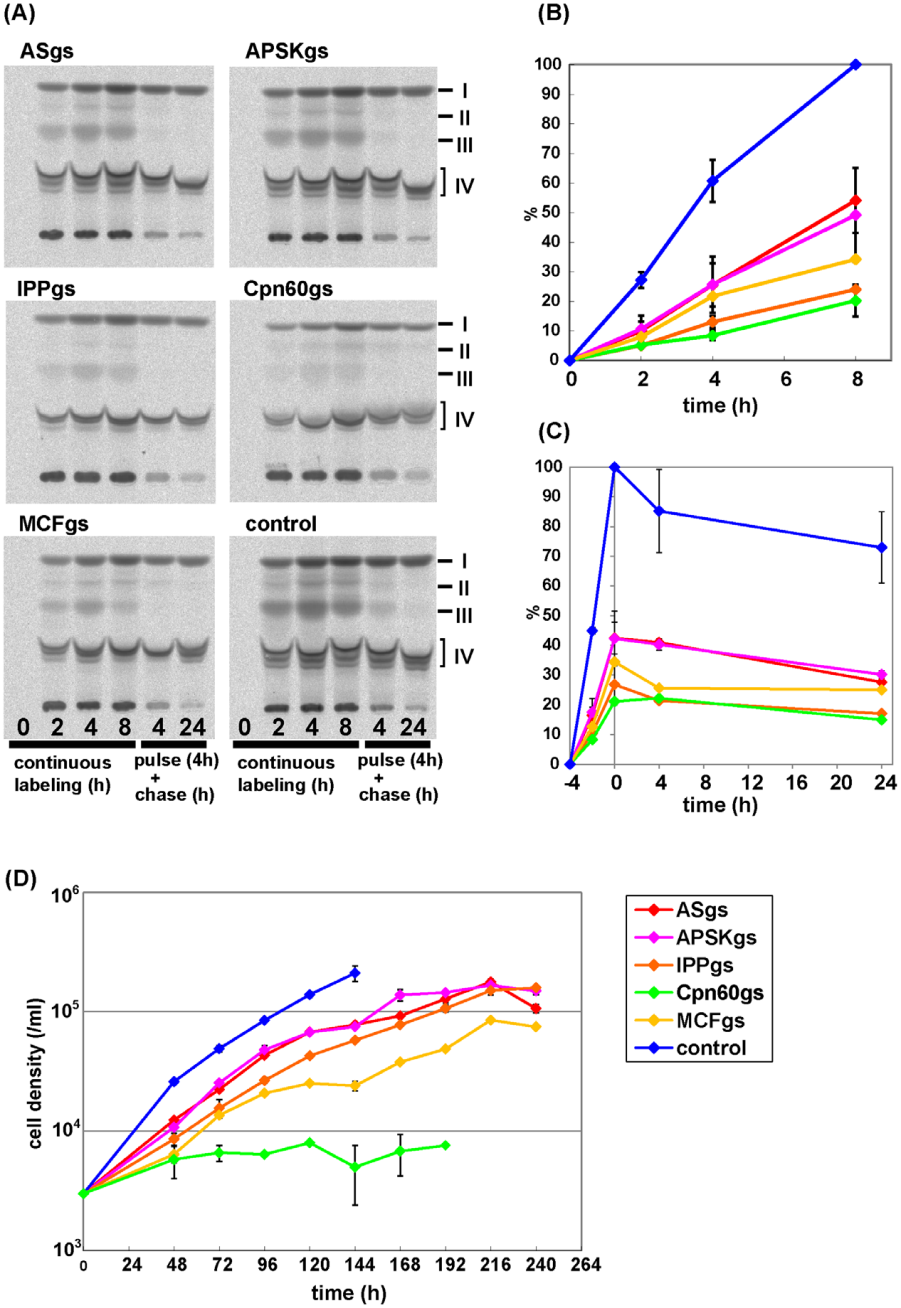
Repression of sulfate activation in the gene-silenced strains. (A) Thin layer chromatography of the extract from the gene-silenced strains. The trophozoites were either continuously cultured in the medium containing ^35^S-labeled sulfate for 0–8 h (4 left lanes), or cultured with ^35^S-sulfate for 4 h and subsequently cultured in the isotope-free medium for 4 or 24 h (2 right lanes). Four major groups of sulfolipids are labeled (“I–IV”). (B–C) Quantitation of total sulfolipid synthesis. The kinetics of the total count of ^35^S incorporated into the sulfated polar lipids that were separated by thin layer chromatography, and measured by densitometric analysis using an image analyzer (Fuji), are shown. The trophozoites were either continuously cultured in the medium containing ^35^S-labeled sulfate for 8 h (B), or cultured with ^35^S-sulfate for 4 h and subsequently cultured in the isotope-free medium for 24 h (C). The level of incorporated ^35^S-sulfate at each point was normalized with protein concentrations and expressed as relative values to the level of ^35^S incorporated in the control strain at 8 h (in continuous labeling experiments, B) or at 0 h (in pulse-chase experiments, C) as 100%. (D) Growth kinetics of the gene-silenced strains. Control; G3 strain transfected with the empty vector (psAP-2-Gunma).

We previously showed that in *E. histolytica* trophozoites, the majority of activated sulfate are incorporated into sulfolipids [5]. So, we first examined the time course of accumulation of sulfolipids in ASgs, APSKgs, IPPgs, and control mock transformants (G3 strain transfected with an empty vector), by metabolic labeling. As shown in Figure 2A, in the control mock transformant, four major groups of sulfolipids (I-IV) were detected by thin layer chromatography, similar to HM1 reference strain as described previously [5]. The amount of sulfolipids (I–IV) changed differently in the individual gene-silenced strains. However, the trend of the decrease of each sulfolipid was similar among the strains: II and III were highly affected, whereas I and IV were not affected as muh as II and III. Thus, only the total count of labeled sulfated lipids is shown. At 8 h of continuous labeling with [^35^S]-sulfate, the sulfate activation activity in ASgs, APSKgs, and IPPgs was decreased to 54.1±11.0, 49.1±15.9, and 24.0±0.6%, respectively, as compared to the control (Figure 2B). We also examined the stability of the accumulated products by pulse-chase experiment. The degradation kinetics of all transformants was similar (Figure 2C). These results indicate that AS, APSK, and IPP are indeed involved in sulfate activation.

We next examined the growth of the gene-silenced strains. Although ASgs, APSKgs, IPPgs, and the mock control showed similar growth pattern, the three former strains showed marked growth retardation. The doubling time of ASgs, APSKgs, and IPPgs was 24.9±2.7, 26.1±2.0, and 34.4±1.2 h, respectively, while that of the control was 15.0±0.8 h (Figure 2D). The degree of the growth inhibition was parallel to the level of repressed sulfate activation activity (Figure 2B, growth rate: IPPgs<APSKgs = ASgs<control). These results indicate that sulfate activation is important for cell proliferation.

Chlorate (ClO_3_^−^), a known inhibitor for AS in mammals and fungi [23]–[25], inhibited cell growth of *E. histolytica* (IC_50_ = 10.5 mM). At this concentration, the sulfate activation activity (as expressed as the total count of labeled sulfated lipids) was decreased to 22.5% of the control. Furthermore, the apparent IC_50_ of the recombinant *E. histolytica* AS by chlorate was determined to be 3.55±0.25 mM. These results support the premise that sulfate activation plays an important role for proliferation.

### Effects of repression of Cpn60 and MCF on sulfolipid production and growth

We also investigated the role of Cpn60 and MCF in sulfate activation. In Cpn60gs and MCFgs strains, the activity of sulfate activation was decreased to 20.2±5.4 and 34.2±1.1%, respectively, as compared to the control (Figure 2B). MCFgs and Cpn60gs strains also showed marked growth defect; the doubling time of MCFgs and Cpn60gs strains was 41.7±5.5 and 82.4±14.5 h, respectively (Figure 2D). These results indicate that MCF and Cpn60 significantly contribute to sulfolipid synthesis and cell proliferation. However, as growth of these gene-silenced strains was moderately-to-severely affected, the growth retardation should be taken into account for the impaired sulfolipid synthesis in these strains.

### Localization and topology of the enzymes in sulfate activation pathway

**Figure 3:**
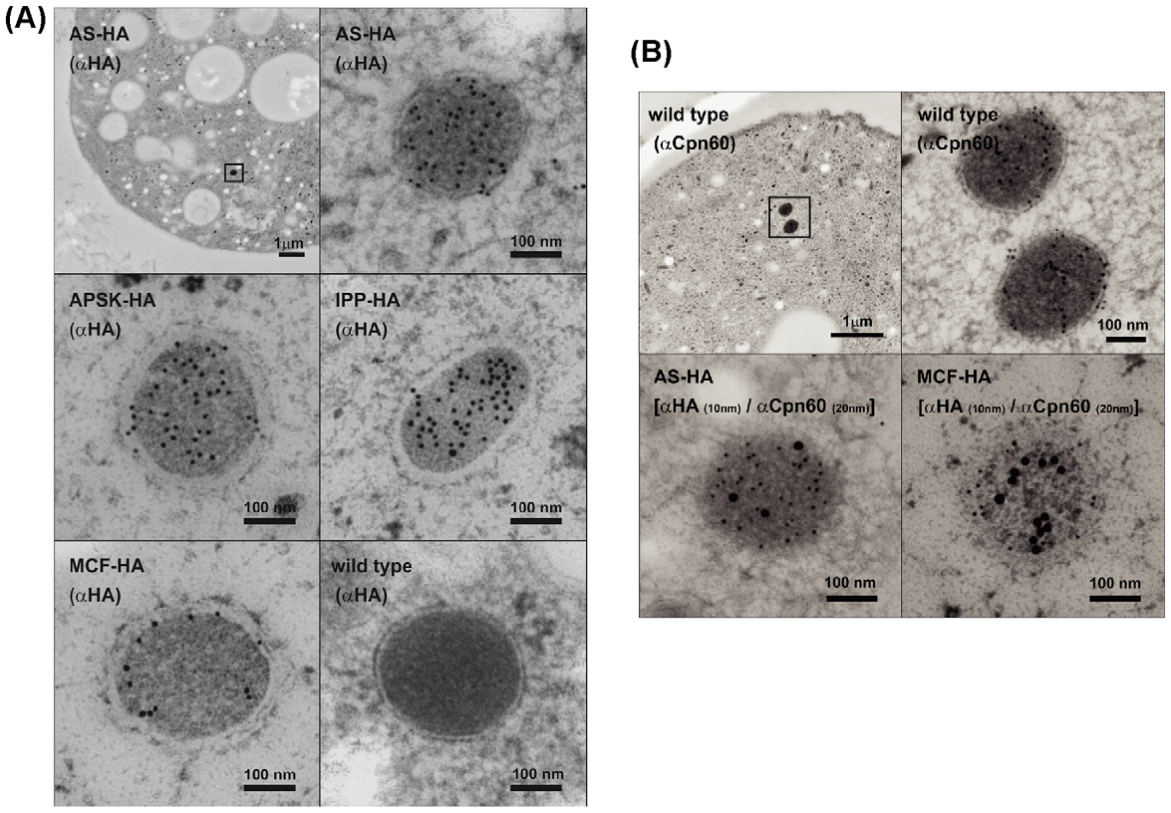
Localization of mitosomal proteins by immunoelectron microscopy. (A) Single labeling of AS, APSK, IPP, and MCF in the transformants that express HA-tagged proteins. Low magnification image is also shown for AS. No labeling was observed in the untransfected cells (“wild type”). (B) Co-localization of AS and MCF with Cpn60. Single staining of Cpn60 (upper panels) and double-labeling of AS and Cpn60 (bottom, left) and MCF and Cpn60 (bottom, right) labeled with colloidal gold particles of different sizes in the transformants expressing HA-tagged AS and MCF.

We investigated the localization and topology of the proteins involved in sulfate activation pathway by exploiting the transformants expressing the HA-tagged proteins (AS, APSK, IPP, and MCF). Immunoelectron microscopy revealed that all the proteins examined are confined to the electron dense double-membrane-surrounded organelles, the size of which are 150–400 nm in diameter (Figure 3A). Double labeling with anti-HA and anti-Cpn60 antibodies showed that these proteins were co-localized with Cpn60, the authentic marker of mitosomes (Figure 3B; only AS and MCF were shown). While AS, APSK, IPP, and Cpn60 were evenly distributed throughout the luminal (matrix) part of mitosomes, MCF was concentrated on the inner membrane of mitosomes. The quantification results for the distribution of AS, APSK, IPP, MCF, and Cpn60 were summarized in Table 1. These proteins were found to be 140-560-fold concentrated in mitosomes as compared to the cytosol. The number of mitosomes was estimated to be 32.1±9.7 per section (10 sections of 10 cells were examined). We estimated by the method described previously [12] that the mitosomes density is about 1.57 per µm^3^, the number of mitosomes per trophozoite is about 6585, and the volume percentage of mitosomes is about 1.2%, in the *E. histolytica* transformant lines used in this study.

**Table 1:** Distribution and density of the mitosomal proteins.

		Mitosomal proteins				
Parameter		Cpn60	AS	APSK	IPP	MCF
Labeling density (golds µm^−2^)	Cytosol	2.13±1.15	2.86±2.12	0.930±0.900	0.908±0.744	0.950±0.908
	Mitosomes	372±102	753±151	356±101	509±197	133±95.6
Distribution ratio (mitosome cytosol^−1^)		175	263	383	561	140

The labeling density of gold particles per µm^2^ area was calculated by examining eight representative sections.

## Discussion

In our previous report, we showed that mitosomes of *E. histolytica* uniquely possess sulfate activation pathway, while they have lost most of the functions shared by other aerobic eukaryote mitochondria including TCA cycle, electron transport, oxidative phosphorylation, and β-oxidation of fatty acids [5]. Only three chaperones and two mitochondrial-type transporters aside from the components in the sulfate activation pathway are retained in *E. histolytica* mitosomes [4], [5], [26]. Since the sulfate activation pathway is not typically confined to the mitochondria, and generally present in either the cytosol or plastid in eukaryotes [5], [7], the physiological significance of its compartmentalization in *E. histolytica* remains unknown [5].

In this report, we have provided several lines of direct and indirect biochemical and cell biological evidence that the sulfate activation pathway plays an important role in the production of sulfolipids and the growth of trophozoites. Consistent with this premise, the AS inhibitor, chlorate, inhibited the sulfolipid production in and the growth of *E. histolytica*. Further supporting the specificity of chlorate to AS, two amino acid residues (Asn198 and His201) of *Saccharomyces cerevisiae* AS, which were implicated in the chlorate binding [24], as well as Arg362, which is located in the highly conserved ISGTxxR motif, are well conserved in *E. histolytica* AS (Asn211, His214, and Arg375).

In addition to the importance of the enzymes in the sulfate activation pathway, we demonstrated that MCF, and Cpn60 also play an important role in cell proliferation. The phenotype of Cpn60gs is most likely attributable to multiple defects as Cpn60 is required for the folding and quality control of mitosome-targeted proteins [27]. Lack of Cpn60 should result in an inability to fold freshly-imported mitosomal proteins and therefore make them functional. This notion likely explains why the knockdown of Cpn60 impaired the cell growth more severely than that of the genes directly involved in sulfate activation. The phenotype of MCFgs strain was probably accounted for the lack of ATP supply required for chaperone functions and for the activation of inorganic sulfate into PAPS in AS- and APSK-catalyzed reactions. The latter possibility was supported by the observation that in MCFgs strain, the activity of sulfate activation was significantly reduced while the amount of the proteins involved in the pathway was not changed. Lack of MCF would impair the ADP/ATP ratio in mitosomes, and, as Cpn60 needs ATP to function, likely cause a similar effect as Cpn60gs. We assume that MCF and Cpn60 are not indispensable for sulfolipid synthesis per se, but gene silencing of these house-keeping proteins resulted in broader effects, and thus severe impairment of mitosome functions.

Immunoelectron microscopy revealed that MCF is mainly localized on the inner mitosome membrane. Although membrane topology may need to be further verified, the observed localization of MCF agrees well with its biochemical characteristics, previously demonstrated: ATP import and ADP export [28]. Recently, *E. histolytica* phosphate transporter (EhPiC) has been identified and proposed to transport phosphate released from ATP through hydrolysis by chaperones, *i*.*e*., Cpn60 and mitochondrial HSP70 [26]. It is conceivable that EhPiC transports phosphate generated by IPP in sulfate activation pathway in mitosomes.

While the structure of *E*. *histolytica* mitosomes revealed by immunoelectron microscopy was far different from the typical eukaryotic mitochondria, it was somehow similar to the mitochondrion-related organelles, described as densely-stained double membrane-bound organelles lacking the typical cristae, in *M*. *balamuthi,* in shape, apparent size, number per cell, and structure [29]. Together with the fact that *M. balamuthi* possesses genes involved in sulfate activation, it is possible that sulfate activation is a unique feature shared only by *E. histolytica* and *M. balamuthi*.

The number and structure of mitosomes demonstrated in this study was slightly different from previous reports [12], [30]. This may be due to the heterogeneity of mitosomes in the cells. We have also previously shown that the distribution of AS, APSK, IPP, MCF, and Cpn60 in mitosomes was not uniform [5], suggesting the heterogeneity of mitosomes in *E*. *histolytica*.
